# Presence of distinctive microbiome in the first-pass meconium of newborn infants

**DOI:** 10.1038/s41598-021-98951-4

**Published:** 2021-09-30

**Authors:** Jenni Turunen, Mysore V. Tejesvi, Niko Paalanne, Jenni Hekkala, Outi Lindgren, Mika Kaakinen, Tytti Pokka, Anna Kaisanlahti, Justus Reunanen, Terhi Tapiainen

**Affiliations:** 1 Medical Research Center and PEDEGO Research Unit, University of Oulu, Oulu, Finland; 2 Biocenter Oulu, University of Oulu, Oulu, Finland; 3 Ecology and Genetics, Faculty of Science, University of Oulu, Oulu, Finland; 4 Department of Pediatrics and Adolescent Medicine, Oulu University Hospital, Oulu, Finland; 5 Cancer and Translational Medicine Research Unit, University of Oulu, Oulu, Finland; 6 Medical Research Center Oulu, University of Oulu, Oulu, Finland; 7 Department of Pathology, Oulu University Hospital, Oulu, Finland

**Keywords:** Microbiome, Paediatric research

## Abstract

We critically evaluated the fetal microbiome concept in 44 neonates with placenta, amniotic fluid, and first-pass meconium samples. Placental histology showed no signs of inflammation. Meconium samples were more often bacterial culture positive after vaginal delivery. In next-generation sequencing of the bacterial 16S gene, before and after removal of extracellular and PCR contaminant DNA, the median number of reads was low in placenta (48) and amniotic fluid (46) and high in meconium samples (14,556 C-section, 24,860 vaginal). In electron microscopy, meconium samples showed extracellular vesicles. Utilizing the analysis of composition of microbiomes (ANCOM) against water, meconium samples had a higher relative abundance of Firmicutes, *Lactobacillus*, *Streptococcus*, and *Escherichia-Shigella*. Our results did not support the existence of the placenta and amniotic fluid microbiota in healthy pregnancies. The first-pass meconium samples, formed in utero, appeared to harbor a microbiome that may be explained by perinatal colonization or intrauterine colonization via bacterial extracellular vesicles.

## Introduction

The fetal microbiome concept was developed after culture-independent methods, mainly next-generation sequencing of the bacterial 16S marker gene, had shown diverse microbiome signatures in the first-pass meconium formed in utero^1–3^, amniotic fluid^1,3^, placenta^1,4–6^, and fetal lungs^7^. The microbiome of meconium has been suggested as a proxy for fetal gut microbiome^8,9^ because it is formed before birth and associates with maternal and environmental factors during pregnancy, such as maternal diet^10–12^, smoking^13^, maternal antibiotics during pregnancy^8^, and the presence of household pets^8,14^ and/or siblings^14^.

Recent high-quality studies have questioned the presence of a distinct placental microbiome^15–20^ and an amniotic fluid microbiome^21,22^. Yet, in animal models, maternal bacteria have been suggested to actively colonize the fetal gut before birth^23,24^. In an interesting study, genetically labeled *Enterococcus*, isolated before the study from a healthy female’s breast milk, was orally administered to pregnant mice^23^. After term Caesarean section (C-section), meconium from fetuses was obtained and labeled bacteria were detected^23^. In humans, maternal mononuclear cells harbor whole bacteria or bacterial antigens frequently during pregnancy^25^.

Contaminant environmental bacterial DNA or DNA from laboratory kits, i.e. kitome, may explain the microbiome findings in samples with a low amount of bacterial DNA^26,27^. In order to critically investigate the proposed fetal microbiome concept further, we set out to characterize microbiomes in the first-pass meconium, placenta, and amniotic fluid; first, by next-generation sequencing of the bacterial 16S gene of unprocessed samples and then after removal of extracellular bacterial DNA and contaminant bacterial DNA in PCR reagents. Additionally, we investigated the histology of the placenta and performed bacterial culture and electron microscopy of the first stool to detect whole-cell bacteria or extracellular vesicles.

## Material and methods

### Study design and study population

**Figure 1:**
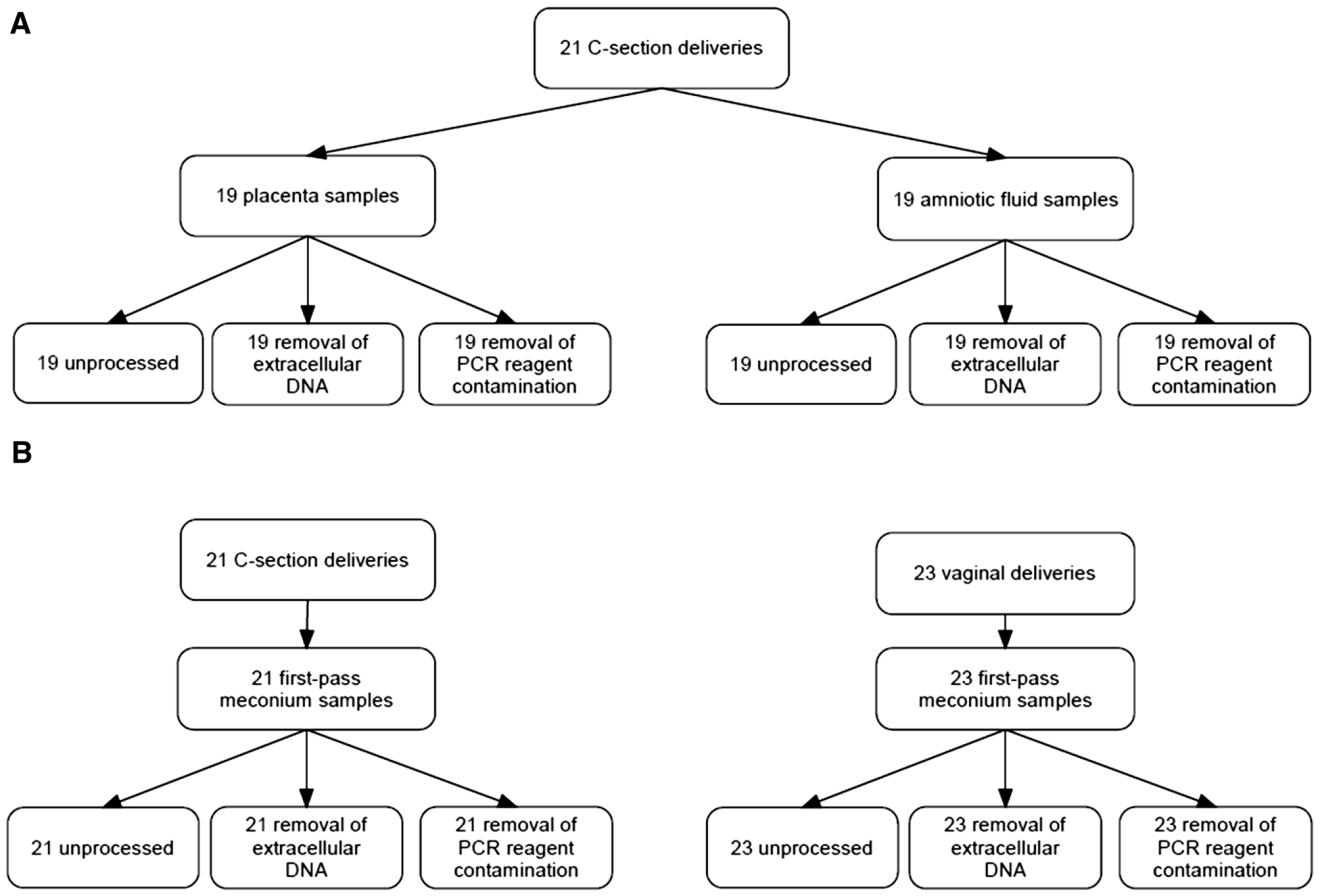
The study design. The study design. In total, samples from 44 newborn infants were used. (**A**) Placenta and amniotic fluid samples. Two C-section deliveries were planned as vaginal deliveries. Due to this, placenta and amniotic fluid samples could not be obtained from those deliveries. (**B**) Meconium samples.

This was a cross-sectional study of 44 term newborn infants born from uncomplicated pregnancies (Fig. 1). Ethical Committee of Northern Ostrobothnia Hospital District at Oulu University Hospital, Finland, approved the study with a decision number EETTMK:3/2016. All families gave their written informed consent before the study, and the experiments were conducted in accordance with all relevant guidelines and regulations. The first-pass meconium, amniotic fluid, and placental samples were split into three subsets: (1) a sample without any processing, (2) a sample processed with a propidium monoazide (PMA) dye set to remove extracellular DNA from samples, and (3) a sample with processed PCR reagents using dsDNase to remove reagent contamination. Thus, each sample was analyzed in triplicate (Fig. 1). For each subset, two negative control samples (sterile water, HyClone™ HyPure, Thermo Fisher Scientific, MA, USA) were prepared using the same protocol.

### Sample collection and bacterial culture

The obstetrician performing the C-section obtained the amniotic fluid samples using a sterile needle through the fetal membranes or by collecting fluid from a sterile kidney dish filled with amniotic fluid during the procedure. None of the amniotic fluid samples were reported to be contaminated by meconium. Data was not available for three samples. Trained midwives collected the placental samples after the C-section. Altogether two 1.5 × 1.5 × 1 cm samples were cut with a sterile knife approximately 3–4 cm away from the cord insertion site (Supplementary Fig. S1). The first-pass meconium, i.e., first stool after birth formed in utero, was collected by either a midwife at the delivery room or the child’s named nurse at the labor ward from a diaper within 24 h from birth. The samples were first placed at − 20 °C and then stored at − 80 °C.

Bacterial culture of the first stool after birth was performed for all meconium samples using routine bacterial culture methods in a clinical microbiology laboratory at Nordlab, Oulu University Hospital, Finland.

### Histology of placenta

Fifteen placentas from C-section deliveries were available for histological evaluation. Representative samples from the cords, membranes, and three full-thickness cross-sections of placental parenchyma were processed according to standard protocol and embedded in paraffin. Histological slides of 3.5 µm thickness were cut from formalin-fixed paraffin-embedded (FFPE) samples and stained using hematoxylin–eosin (Dako CoverStainer, CS100, Agilent, CA, USA) at Oulu University Hospital Pathology Department. Histological evaluation of placental infection was done accordingly to Amsterdam Placental Workshop Group Consensus criteria^28^.

### Electron microscopy and characterization of nanoparticles of the first-pass meconium

Electron microscopy of meconium samples was performed at Biocenter Oulu, Finland, according to the following protocol: approximately 50 µg meconium was suspended in 200 µl sterile phosphate-buffered saline (PBS). Next, 5 µl of this solution was placed on carbon-coated and glow-discharged grid and incubated for 20 min. The grid was then washed for 1 min twice in 100 µl of PBS. The sample was fixed by incubating the grid on a drop of 1% glutaraldehyde in PBS for 5 min. The grid was washed eight times for 1 min in a drop of distilled water and stained with neutral 2% UA in 50 µl water for 5 min. The grid was coated with 50 µl 2% methylcellulose-UA (0.4%) solution for 10 min on ice and removed from the drop with loop. Excess fluid was blotted by pushing the loop sideways on Whatman no. 1 filter paper, leaving thin film on the EV side of the grid. The grid was air-dried for 10 min and stored in a grid storage box until electron microscopy analysis. The imaging was performed using a Tecnai Spirit transmission electron microscope (Fei Europe) and a Quemesa CCD camera (Olympus Soft Imaging Solutions GMBH).

Meconium samples (2 vaginal deliveries and 2 C-sections) were suspended into 1 × Dubelcco’s phosphate buffered saline (dPBS) (100 mg/ml) and centrifuged for 2 min at 3000×*g* to pellet solid material. Supernatant was collected for further analyses by microscopy and Nanoparticle Tracking Analysis (NTA). The size of particles (smaller than 600 nm) and particle concentration were measured by NTA using Nanosight 300 (Malvern). Samples were diluted to 1:13 in 1 × dPBS and loaded to NS300 for imaging. Each sample was captured four times 60 s video and each 1 s frame was analyzed via Nano Tracking Analysis. For light microscopy, the samples were serially diluted 1:10 in dPBS, applied on microscopic slides and allowed to air dry, followed by heat fixation and Gram-staining.

### Removal of extracellular DNA with propidium monoazide

To remove extracellular DNA from the samples, we used propidium monoazide (PMA) dye. We chose PMAxx™ dye (Biotium, CA, United States), which has been used successfully in a similar study^29^. The manufacturer’s protocol was followed. For meconium and placenta samples, 200 mg of sample was dissolved in 1 ml of phosphate-buffered saline (PBS) with bead beating, and 1.25 µl of 25 mM PMA was added. For amniotic fluid samples, 1.25 µl of 25 mM PMA was added to 500 µl of amniotic fluid. Samples were incubated in the dark for 10 min with occasional vortexing and then exposed to light in an ice bath for 15 min. Samples were centrifuged at 5000 rpm for 10 min. Then the pellet was resuspended to DNA extraction buffer, after which the DNA extraction continued with the cell lysis step.

### DNA extraction

DNA was extracted using QIAamp PowerFecal Pro DNA Kit (Qiagen, Germany) according to the manufacturer’s protocol. Meconium and placenta samples were homogenized using bead beating at 25 Hz for 2 min in the TissueLyser (Qiagen) and incubated on ice for 1 min. Steps were repeated 1–3 times. Amniotic fluid samples were centrifuged for 25 min at 13,000 rpm, the supernatant was discarded, and the pellet was dissolved in extraction buffer. After this, DNA extraction was performed. The final DNA elution was set to 50 µl to increase the DNA yield. DNA concentration was measured using NanoDrop 1000 Spectrophotometer (Thermo Fisher Scientific).

### Removal of bacterial DNA contamination in PCR reagents by dsDNase treatment

Before performing PCR, one-third of samples’ PCR master mixes went through dsDNase treatment using a PCR Decontamination Kit (ArcticZymes, Tromsø, Norway) according to the manufacturer’s protocol. The kit has earlier been evaluated and used on PCR reagents to purify them of DNA contaminations on a similar fetal microbiome study^3,30^.

### PCR, 16S rRNA gene sequencing, and analysis

We performed sequencing of the 16S rRNA gene’s V4–V5 region using primer 519F with unique barcodes as well as primer 926R. For PCR, the manufacturer’s protocol of Phusion Flash High-Fidelity PCR master mix (Thermo Fisher Scientific) was followed. A negative control (sterile water, HyClone™ HyPure, Thermo Fisher Scientific) was used in each PCR plate, as well as five positive controls of HM-782D, Microbial Mock Community B (BEI resources, USA) to each sequencing run. PCR was performed with Applied Biosystems™ Veriti 96-Well Thermal Cycler (Thermo Fisher Scientific). The PCR initialization program was ran for 3 min at 98 °C, followed by 30 cycles of reaction, starting at 98 °C for 10 s and followed by 30 s at 56 °C annealing temperature and elongation at 72 °C for 30 s. Final elongation was at 72 °C for 5 min.

The samples were pooled and purified using AMPure XP (Beckman Coulter, CA, USA). The purified pool was run in 1% agarose gel, after which the 16S product was cut from the gel and purified with MinElute Gel Extraction Kit (Qiagen). The purified product went through second PCR with Phusion Flash High-Fidelity PCR master mix using 1 µM HPLC-purified primers A and trP1. The program was run for seven cycles at a 63 °C annealing temperature and 5 min of elongation. The final product was purified with AMPure XP, analyzed with Bioanalyzer, and the concentration of the pool was measured using Quant-iT PicoGreen dsDNA Assay Kit (Thermo Fisher Scientific). The sequencing was performed on IonTorrent PGM (Thermo Fisher Scientific).

Analysis was performed on Quantitative Insights Into Microbial Ecology 2 (QIIME2; version 2020.2 and 2020.6)^31^. DNA reads under 200 bp were omitted from the taxonomic analysis. Reads were then demultiplexed and denoised with DADA2^32^. Denoised reads were trimmed at 15 and truncated at 260, and chimeric reads were filtered out, resulting in a total of 3,994,640 processed reads ready for further analyses. R package decontam (version 1.8.0) was used to filter out environmental contaminants from each sample type using a prevalence-based method with a threshold of 0.5^33^. Furthermore, taxa identified as Cyanobacteria, Mitochondria, Eukaryota, or Archaea were removed, similar to previous fetal microbiome studies^6,12,17,34,35^. Reads were normalized using R package MetagenomeSeq (version 1.30.0)^36^. Samples with less than 10 reads and taxa that were not present in the samples were removed. Reads were normalized with a normalization percentile calculated using the cumNormStat function.

We performed Principal Coordinate Analysis (PCoA) with Bray–Curtis dissimilarity and taxonomic analysis of the samples at phylum and genus levels. Taxonomic compositions in each sample group were calculated by relative frequency. The SILVA database (version 138) was used for the taxonomic analysis^37^. Figures were drawn on RStudio^38^ using packages ggplot2 (version 3.3.5)^39^, grid (version 4.0.2)^38^, and GridExtra (version 2.3)^40^.

### Statistical analysis

We employed analysis of composition of microbiomes (ANCOM) using the QIIME2 bioinformatics platform and Mann–Whitney U test, confirmed by p-test, applying RStudio for the differential abundance analysis of all taxa in the samples^41^. Beta diversities’ statistical analyses were calculated using PERMANOVA and confirmed with a p-test. The proportions of bacterial culture-positive samples were compared using the StatsDirect analysis for two proportions.

## Results

### Study population

In total, 44 term newborn infants from uncomplicated pregnancies were enrolled in the study (Table 1). Altogether 23 infants were vaginally delivered and 21 were born via C-section. Bacterial culture of the first-pass meconium was positive in 16/22 (73%) of vaginally born newborn infants and 3/19 (16%) of those born via C-section (p < 0.001, 95% confidence interval of the difference 27% to 76%; Table 2). All placental samples were morphologically normal with normal terminal villi development. None met the diagnostic histological criteria for acute chorioamnionitis, subchorionitis, or chorionitis, i.e. no maternal inflammatory response was observed. No detectable funisitis, chorionic vasculitis or umbilical phlebitis was observed, i.e. no signs of fetal inflammatory response were seen. No detectable villitis or chronic basal plate inflammation was observed.

**Table 1:** Characteristics of the study participants.

	Vaginal N = 23	C-section N = 21^a^	All N = 44
Mothers age, year mean (SD)	28.3 (5.0)	35.8 (5.0)	31.9 (6.3)
Gestational age (weeks) mean (SD)	39.5 (2.3)	39.3 (0.9)	39.4 (1.8)
Birth weight (grams) mean (SD)	3510 (280)	3740 (620)	3620 (480)
Boys (%)	12 (52%)	10 (48%)	22 (50%)
Maternal asthma	0	4 (19%)	4 (9.1%)
Maternal allergy	8 (35%)	11 (52%)	19 (43%)
Gestational diabetes	4 (17%)	8 (38%)	12 (27%)
*Streptococcus agalactiae*^b^	5 (22%)	9 (43%)^b^	14 (32%)
Antibiotics during pregnancy	9 (39%)	5 (24%)	14 (32%)*
Intrapartum antibiotics	5 (22%)	21 (100%)	26 (59%)**
Perinatal antibiotics after birth	1 (4.3%)	1 (4.8%)	2 (4.5%)
NICU admission	1 (4.3%)	2 (9.5%)	3 (7.0%)

*NICU* neonatal intensive care unit.

^a^2 patients recruited in the vaginal delivery group gave birth via C-section and were analyzed as such.

^b^*Streptococcus agalactiae* screening before birth was not performed in 6 cases.

*In the vaginal delivery group, 1 mother had been administered amoxicillin. In the C-section group 1 mother had been administered amoxicillin and 1 received cephalexin.

**In the vaginal delivery group, all 5 mothers had been administered benzylpenicillin. In the C-section group, 19 mothers had been administered cefuroxime, 1 Piperacillin-Tazobactam, and 1 received Clindamycin.

**Table 2:** Bacterial culture of the first-pass meconium.

	Vaginal N = 23	C-section N = 21	All N = 44
Negative	7 (30%)	16 (76%)	23 (52%)
Coagulase-negative staphylococci	6 (26%)	2 (9.5%)	9 (20%)
*Escherichia coli*	3 (13%)	0	3 (6.8%)
*Lactobacillus* species	2 (8.7%)	0	2 (4.5%)
Group viridans streptococci	1 (4.3%)	1 (4.8%)	2 (4.5%)
*Staphylococcus epidermidis*	1 (4.3%)	0	1 (2.3%)
*Bacillus* species	1 (4.3%)	0	1 (2.3%)
*Propionibacterium* species	1 (4.3%)	0	1 (2.3%)
Group B streptococci	1 (4.3%)	0	1 (2.3%)
Culture results unavailable	1	2	3

### Basic microbiome analysis

**Figure 2:**
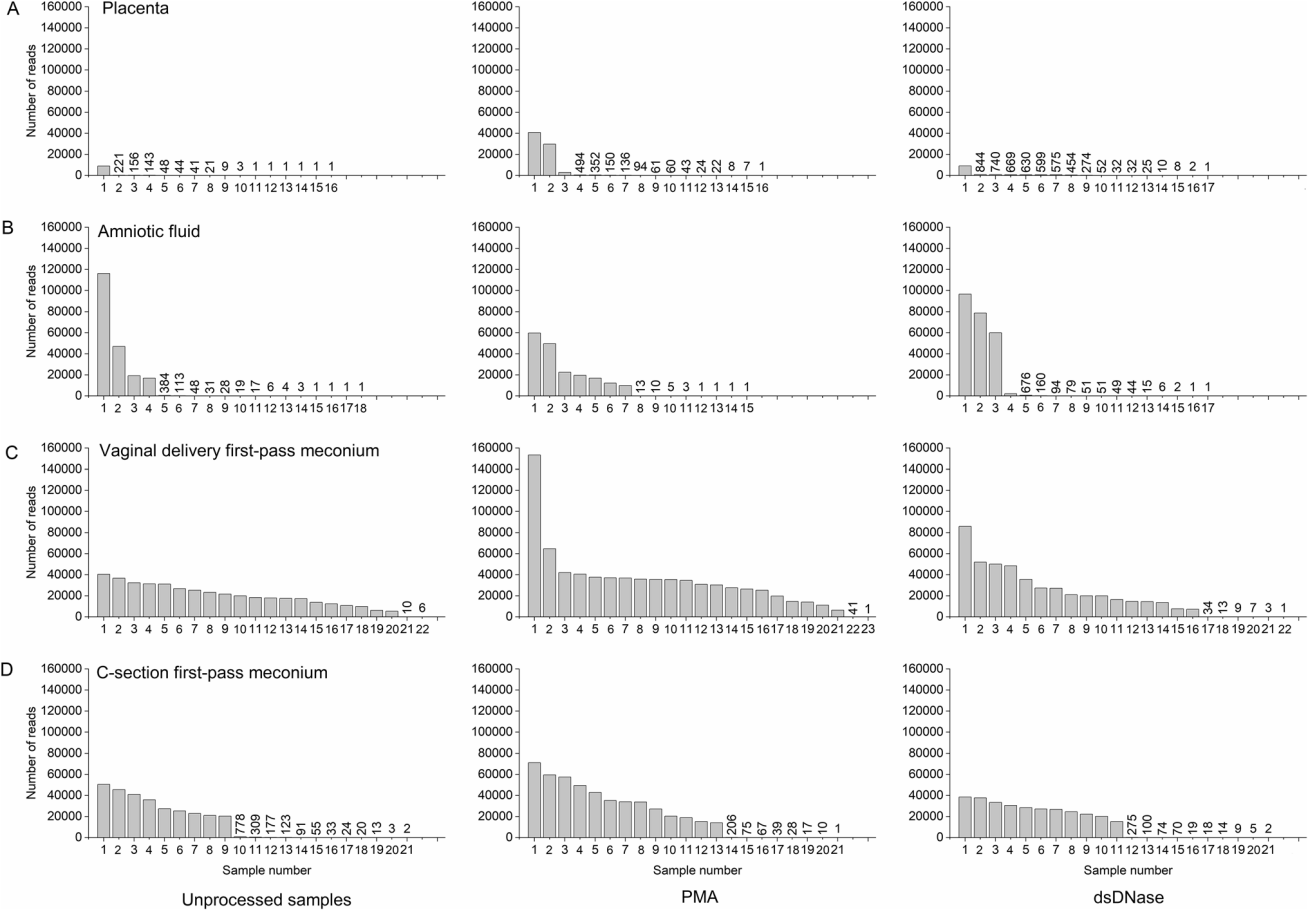
Read counts of raw sequencing data according to sample types (**A–D**) and laboratory processing. PMA indicates propidium monoazide used to remove extracellular DNA, and dsDNase indicates the decontamination of PCR reagents. Each column indicates one sample. (**A**) Placental samples without processing, after PMA, and after dsDNase treatments (**B**) Amniotic fluid samples without processing, after PMA, and after dsDNase treatments. (**C**) First-pass meconium samples after vaginal birth without processing, after PMA, and after dsDNase treatments. (**D**) First-pass meconium samples after C-section delivery without processing, after PMA, and after dsDNase treatments*.*

Read counts of raw data varied markedly between sample types (Fig. 2). The majority of placenta (30/49, 61%) and amniotic fluid (31/50, 59%) samples had fewer than 100 reads. In total 37% (23/63) of meconium samples after C-section had fewer than 100 reads, whereas 15% (10/67) of those after vaginal delivery had < 100 reads. The median number of reads was low in placenta (48) and amniotic fluid (46) and high in meconium samples (14,556 C-section, 24,860 vaginal). The variation was less clearly seen between the processing groups, including PMA treatment to remove extracellular DNA and dsDNase treatment to remove bacterial contamination from PCR reagents (Fig. 2). Alpha diversity analysis concluded no significant difference in observed operational taxonomic units (OTUs) in placenta and amniotic fluid samples versus the negative control (sterile water) except for unprocessed placenta samples, where water samples, in fact, contained more observed OTUs, and dsDNase-treated amniotic fluid samples (Supplementary Table S1).

### Principal coordinate analysis

**Figure 3:**
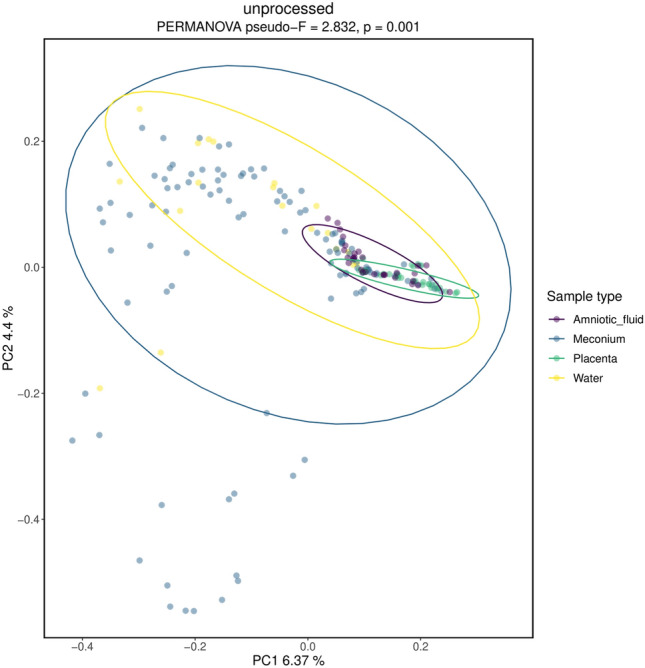
Principal Coordinate analysis using Bray–Curtis dissimilarity. PERMANOVA was used as a statistical test. The significance was confirmed via a p-test.

In Principal Coordinate Analysis using Bray–Curtis dissimilarity, significant differences between samples and controls were observed (p = 0.001; Fig. 3). Placenta and amniotic fluid samples were closely clustered near the negative control samples whereas highly diverse meconium samples clearly differed from the placenta, amniotic fluid, or negative control samples.

### Analysis of composition of microbiomes

ANCOM was performed on all sample types and water, as well as all samples with different treatments within each sample type (Table 3). Placenta and amniotic fluid had two differentially abundant taxa against water: Actinobacteriota and *Cutibacterium.* In meconium against water, differentially abundant taxa included Firmicutes, *Lactobacillus*, *Streptococcus*, and *Escherichia-Shigella* group. Different treatments did not yield differentially abundant taxa in any sample type aside from *Streptococcus* in placenta.

**Table 3:** ANCOM results including differentially abundant OTUs in all sample groups.

	OTU	W
Placenta *vs.* water	*Cutibacterium*	172
Placenta, unprocessed *vs.* PMA *vs.* dsDNase	*Streptococcus*	16
Amniotic fluid *vs.* water	Actinobacteriota	8
	*Cutibacterium*	180
Amniotic fluid, unprocessed *vs.* PMA *vs.* dsDNase	*Methylobacterium*	95
Meconium *vs.* water	Firmicutes	22
	*Lactobacillus*	547
	*Streptococcus*	533
	*Escherichia-Shigella*	500

### Microbiome of the first-pass meconium

As amniotic fluid and placenta samples were unlikely to possess a distinct microbiome based on the very low number of reads, observed OTUs, Bray–Curtis dissimilarity, and ANCOM analysis, we continued with a detailed analysis of the first-pass meconium samples.

**Figure 4:**
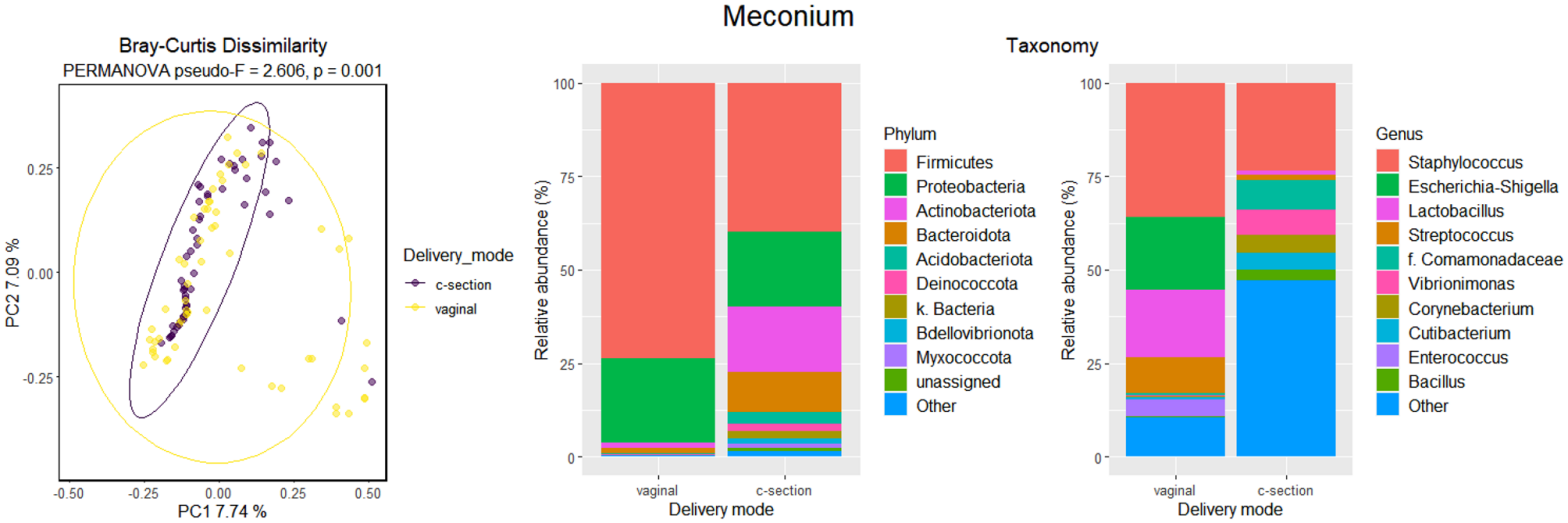
Beta and taxonomy analysis of the first-pass meconium samples, according to the delivery mode. The results of Bray–Curtis dissimilarity and PERMANOVA analysis are shown. Relative abundances of OTUs are presented at phylum and genus levels. The ten most abundant phyla and genera are shown.

When all meconium samples were divided based on delivery mode, significant differences were found between the microbiome compositions of meconium after vaginal and C-section deliveries (Fig. 4). The microbiomes of meconium samples after C-section formed a tight cluster, whereas the microbiomes of the samples obtained after vaginal delivery showed diverse patterns (Bray–Curtis Dissimilarity (p = 0.001; Fig. 4). Firmicutes was the most abundant phylum in both samples after vaginal and C-section deliveries, followed by Proteobacteria (Table 4). Significant differences were found in phyla Actinobacteria (p = 0.008) and Firmicutes (p = 0.001, W = 27), as well as several genera between delivery modes (Supplementary Table S2).

**Table 4:** Ten most abundant species in the first-pass meconium according to the delivery mode.

Phylum	Vaginal (%)	C-section (%)	Genus	Vaginal (%)	C-section (%)
Firmicutes	74	40	*Staphylococcus*	36	23
Proteobacteria	22	20	*Escherichia-Shigella*	20	0.2
Actinobacteriota	1.3	18	*Lactobacillus*	18	1.0
Bacteroidota	1.6	11	*Streptococcus*	9.7	1.5
Acidobacteriota	0.3	2.9	f. Comamonadaceae	0.4	7.9
Deinococcota	0.2	2.2	*Vibrionimonas*	0.3	6.7
K. Bacteria	0.01	1.7	*Corynebacterium*	0.4	4.7
Bdellovibrionota	0.01	1.5	*Cutibacterium*	0.4	4.5
Myxococcota	0.03	1.3	*Enterococcus*	4.5	0.2
Unassigned	0.03	0.8	*Bacillus*	0.3	2.7
Other	0.2	1.4	Other	11	47

**Figure 5:**
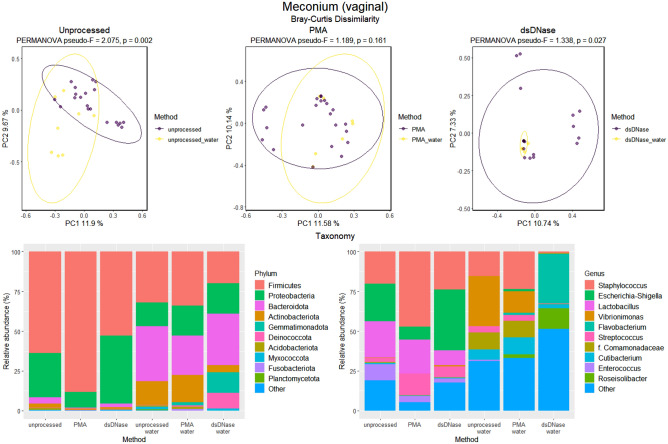
Beta and taxonomy analysis of the microbiome in meconium after vaginal delivery. The three laboratory methods used: Unprocessed samples, PMA treatment to remove extracellular DNA, and dsDNAase treatment to remove bacterial contaminant DNA from PCR reagents. The results of Bray–Curtis dissimilarity and PERMANOVA analysis are shown. Relative abundances of OTUs are presented at phylum and genus levels. The ten most abundant phyla and genera are shown. Water was used as a negative control.

**Figure 6:**
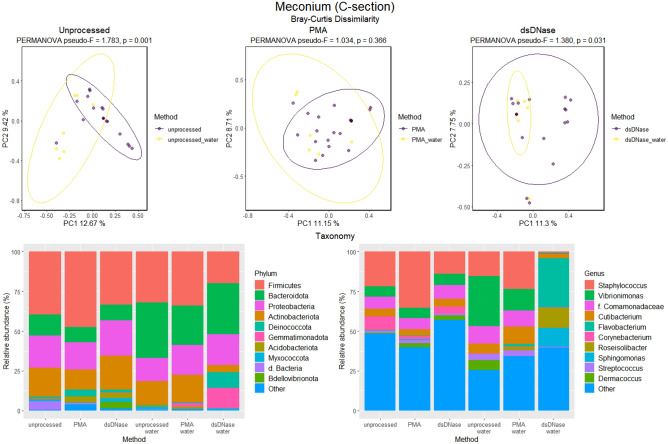
Beta and taxonomy analysis of the microbiome in meconium after C-section. The three laboratory methods used: Unprocessed samples, PMA treatment to remove extracellular DNA, and dsDNAase treatment to remove bacterial contaminant DNA from PCR reagents. The results of Bray–Curtis dissimilarity and PERMANOVA analysis are shown. Relative abundances of OTUs are presented at phylum and genus level. The ten most abundant phyla and genera are shown*.* Water was used as a negative control.

To elucidate better the characteristics of the microbiome in meconium, we then compared the microbiomes of meconium samples according to different laboratory treatments performed to remove contaminant bacterial DNA. The meconium microbiome differed statistically significantly from water in both the vaginal and C-section groups with and without dsDNase treatment (Figs. 5, 6). Following PMA treatment to remove extracellular bacterial DNA, the differences were not statistically significant (Figs. 5, 6). The number of observed OTUs was significantly greater in vaginally delivered meconium samples compared with the control samples (sterile water; Supplementary Table S1). C-section delivery samples had significantly more OTUs exclusively in the dsDNase treatment group (Supplementary Table S1).

### Electron microscopy of the first-pass meconium

**Figure 7:**
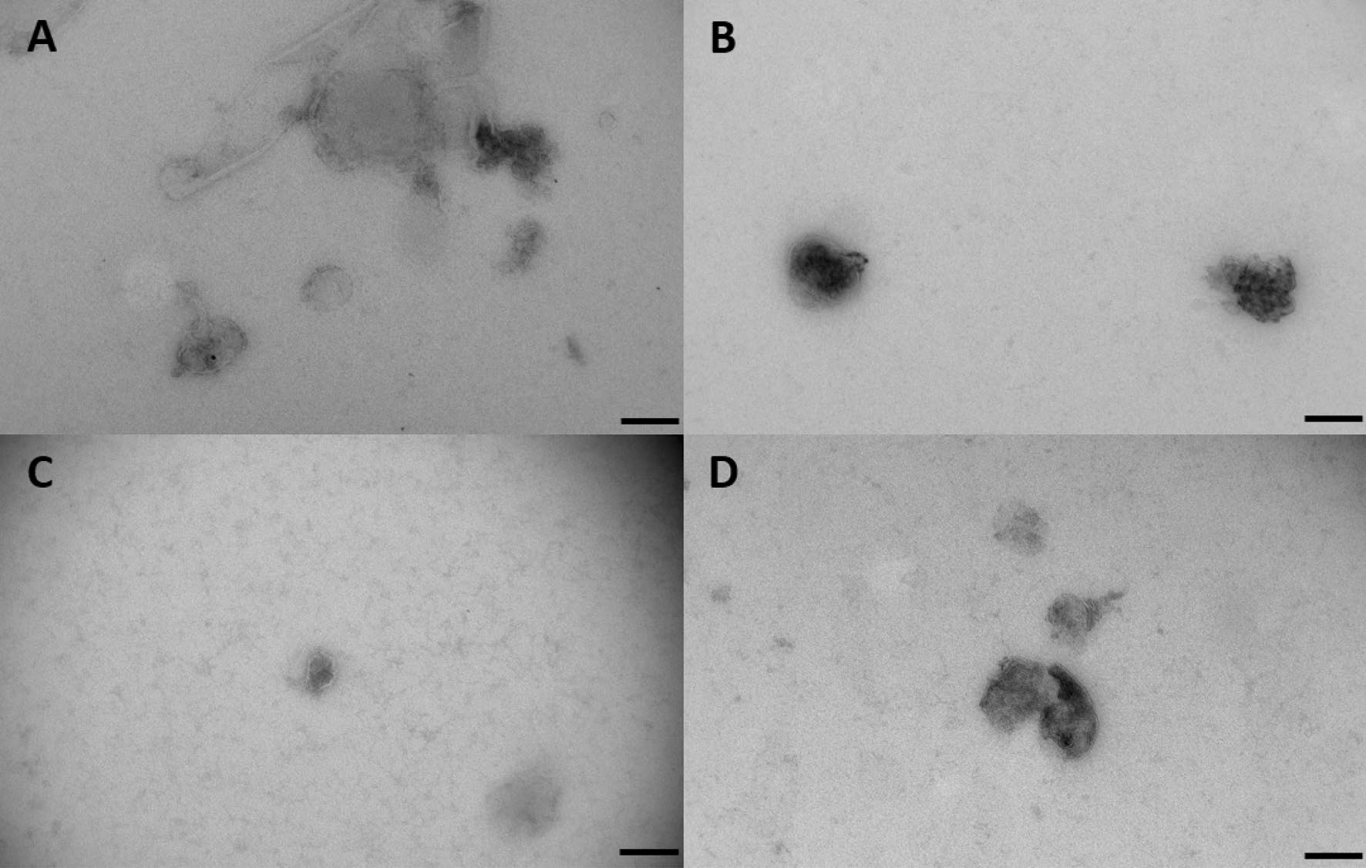
Electron microscopy figures of four different meconium samples. Two samples were from C-section delivery and two from vaginal delivery. Extracellular vesicles are visualized in all first-pass meconium samples both after vaginal delivery and C-section. (**A,B**) C-section delivery sample. (**C,D**) vaginal delivery sample. The size of the scale bar is 200 nm.

We then characterized whether whole bacterial cells or extracellular vesicles were present in the first-pass meconium using electron microscopy of six samples in total. Electron microscopy of the first-pass meconium showed extracellular vesicles in samples both after vaginal and C-section deliveries (Fig. 7). Vesicle sizes ranged from approximately 100 nm to 200 nm in diameter.

### Characterization of nanoparticles

In microscopic analysis of the nanoparticles, only saffranin-stained particles not equalling in size and shape with bacterial cells could be observed. This indicated intact Gram-positive bacterial cells being absent or very low in numbers in the meconium samples, whereas fairly high amounts (5.0 + E09 − 1.378 + E10 particles/g) of particles falling into the size range of EVs (20–600 nm) could be detecteced by the NTA.

## Discussion

In the present study, aligning with earlier findings^17^, we could not confirm the presence of the placenta and amniotic fluid microbiome. The first-pass meconium, however, appeared to harbor an actual microbiome, which may be explained by perinatal colonization, or hypothetically, partly by intrauterine colonization through bacterial extracellular vesicles, based on preliminary electron microscopy findings.

Our results do not support the idea of an existing microbiota in amniotic fluid or placenta. This aligns with the results from earlier high-quality studies^15,18,19,42^. Our results, however, showed that first-pass meconium likely harbors a distinct microbiota. We found that microbiomes of the meconium samples obtained after C-section delivery differ from those after vaginal delivery, which is analogous to the results of earlier studies^11,43–45^. This may indicate that meconium already shows the first steps of actual perinatal gut colonization. This idea is supported by the present study’s bacterial culture results showing that most meconium samples after vaginal delivery yielded positive cultures. Furthermore, the number of reads was markedly higher in the first-pass meconium samples than in amniotic fluid or placenta samples, the number of reads being highest in the meconium samples after vaginal birth. Finally, meconium samples seemed to harbor bacteria known to be the first colonizers of the gut, such as *Staphylococcus, Lactobacillus*, *Escherichia*, and *Enterococcus*.

One possible mechanism of perinatal colonization was presented by Lannon et al*.*, who concluded in an interesting study that lactobacilli and bacterial vaginosis-associated bacteria can ascend from the vagina to the chorioamnion during term labor even in the absence of chorioamnionitis^46^. This may be one mechanism of bacteria inhabiting meconium during birth. In an earlier study, maternal gut microbes were found to transport to breast milk via mononuclear cells, potentially developing a neonate’s immune system during feeding^25^. Similarly, microbes might be transported from the mother to the fetus inside cells. In a more recent study by Kennedy et al*.*, meconium samples were collected from C-sectionally delivered children after sectioning but before delivery, thus reducing the risk of contamination and transmission of bacteria from the mother to children during delivery^47^. Contrary to previous findings, this study did not find a distinctive meconium microbiota before birth^47^, which supports the idea of initial gut colonization occurring during and/or after birth.

There is a possibility that differences found between the delivery modes in meconium may be partly affected by the usage of maternal antibiotics. In our study population, 100% of the mothers giving birth via C-section received intrapartum antibiotic treatment, whereas from vaginally delivering mothers only 22% received antibiotics. It has been previously shown that maternal antibiotic usage may affect the neonatal gut development^8,45^. It is possible that the antibiotic treatment has elevated the differences in meconium microbiota of C-sectionally delivered neonates. However, in an earlier study by Tapiainen et al*.*, the effect of perinatal antibiotics couldn’t be seen in newborns until 1–2 days after birth^48^. Furthermore, in this study cohort, mothers in the C-section group had more reported health conditions than those delivering vaginally. These conditions affect the microbiota of the mother, and there are implications of maternal health conditions affecting the child’s microbiota via bacterial transfer^49^.

An important consideration in the meconium microbiome studies is the sampling time. Meconium is often defined as the first stool passed within 48 h from birth. Neonatal microbiota starts diversifying quickly after birth, and it is expected that longer sampling times may cause samples to contain bacteria obtained after birth. However, in an earlier study it was concluded that sampling times of 24 h and less after birth did not affect the bacterial load of the samples^8^. Therefore, it is unlikely that the sampling time affected these results in the present study.

Bacterial contamination from the environment or laboratory kits and reagents have been suggested as explanations for earlier findings of proposed fetal microbiome in the amniotic fluid and placenta in healthy pregnancies^26,34^. In the present study, we aimed to solve this problem by carefully removing bacterial DNA contamination and non-viable bacteria based on the methods used in previous fetal microbiome studies^29,30^. We chose to use PMA and dsDNase to exclude non-cellular DNA from samples and remove contaminant DNA from PCR reagents. The impact on findings from the placenta and amniotic fluid samples was smaller than expected because even unprocessed samples showed very low read counts (Fig. 3) and few OTUs (Supplementary Table S1).

Previous studies about the microbiome of the first-pass meconium have mainly focused on sequencing of bacterial 16S gene in meconium^1–3,8,10,11,43,50–52^. In the present study, we first performed electron microscopy analysis and then the characterization of nanoparticles of the first-pass meconium. We detected few whole bacterial cells in meconium samples using electron microscopy. Using nanoparticle Tracking Analysis, we found many smaller particles fitting the size range of EVs. EVs are small particles secreted from either cell membranes or from endosomes^53^. They are known carriers of various molecules, such as RNA and DNA, and able to cross biological barriers^54^. It is known that both gram-positive^54,55^ and gram-negative bacteria^56^ are able to secrete what is called membrane vesicles (MVs) and outer-membrane vesicles (OMVs) that carry functional genetic material. Hypothetically, these bacterial EVs could contribute to the colonization of the fetus in utero. We did not verify whether EVs were of bacterial or human origin in this study. Thus, our findings remain speculative about the intrauterine colonization process by bacterial EVs derived from the maternal microbiome. Yet, the idea of fetal microbial EV contacts is both intriguing and plausible due to their barrier-crossing abilities. In the future, this novel hypothesis should be further investigated.

The first-pass meconium is the first readily available sample for gut microbiome studies after birth and may reflect the first steps of true bacterial colonization of the gut. It appeared that the differences in the gut microbiome development between newborn infants born via vaginal route and C-section are detectable already in the first stool after birth. Thus, the recently suggested interventions for changing gut colonization in newborn infants born via C-section, such as fecal transplant from the mother’s first milk^57^, may already be late if the very first steps of that colonization process are crucial for later health. Yet, the clinical impact of the microbiome of meconium is still poorly understood. In our earlier prospective cohort studies, using an earlier cohort of consecutive newborn infants, the microbiome of meconium has been associated with subsequent infantile colic^51^ and overweight^52^.

The present study has several strengths. We used a wide array of methods to characterize the fetal microbiome concept in a meaningful way. We compared different sample types, including placenta, amniotic fluid, and the first-pass meconium samples, used different laboratory methods in processing the samples for 16S sequencing to remove bacterial contamination, used histological evaluation of placentas to exclude chorioamnionitis, and performed bacterial culture and electron microscopy of meconium in addition to routine 16S sequencing studies. Furthermore, we had a sufficiently sized negative control group going through the same 16S sequencing protocol. The importance of negative controls is generally recognized, but the number of negative controls is often limited in earlier studies, thus reducing the power of the negative control analysis. We ensured a sufficient number of negative controls (24) in each step of the study. Especially in studies with very low-biomass material, a negative control sample group is essential.

Our study has several limitations. First, sequencing only one small region of the 16S rRNA gene limits bacterial identification up to the species level. Sequencing the entire gene would most likely increase the number of taxa found in samples and accurately assign names to species. Furthermore, choosing the gene’s region and primers may slightly affect the results. Primers have different biases towards and against different taxa, which may result in excluding important taxa present in the samples^58^. Second, samples were most likely slightly contaminated during the entire workflow. This is inevitable, as it is impossible to work in a completely sterile space from start to finish. We avoided as many environmental contaminants as possible by working in a cleaned-up laminar airflow, using sterile instruments, and avoiding any splashing from a sample to another, as well as using Decontam to computationally remove environmental contaminants from our sequence data. However, there is still a possibility of cross-contamination, especially during PCR, when the neighboring wells are close together. In an ideal situation, samples would be positioned in the plate so that neighboring wells would be left empty to avoid the so-called splashome^34^. This approach is often not realistic for a large sample size due to the cost and time limitations. Third, the intrapartum antibiotic treatment and underlying medical conditions of mothers may have contributed to the differences found in meconium microbiome according to delivery modes.

We conclude that the placenta and amniotic fluid appeared to not harbor a unique microbiota. However, a distinct microbiota appears to be likely present in meconium, the first stool after birth formed in utero. Perinatal colonization appeared to play a role in the development of meconium microbiota since its composition depended on the delivery mode. Hypothetically, some bacterial DNA may have been transferred from mother to fetus in utero via extracellular vesicles, based on preliminary electron microscopy findings on meconium. This idea warrants further research.

## Supplementary Information

Supplementary Information.

## Data Availability

The raw sequences that support the findings of this study are available in sequence reads archives (SRA) with a BioProject accession number PRJNA691124.
