# Supplementary material for: Presence of distinctive microbiome in the first-pass meconium of newborn infants
Source: Sci Rep. 2021 Sep 30;11:19449. doi: 10.1038/s41598-021-98951-4 (PMC8484610; doi:10.1038/s41598-021-98951-4)
Supplement: Supplementary file 1 — Supplementary Information. [file 41598_2021_98951_MOESM1_ESM.pdf]

Supplementary information

Supplementary figure S1. The sampling sites of the placental samples are delineated by black squares in the figure.

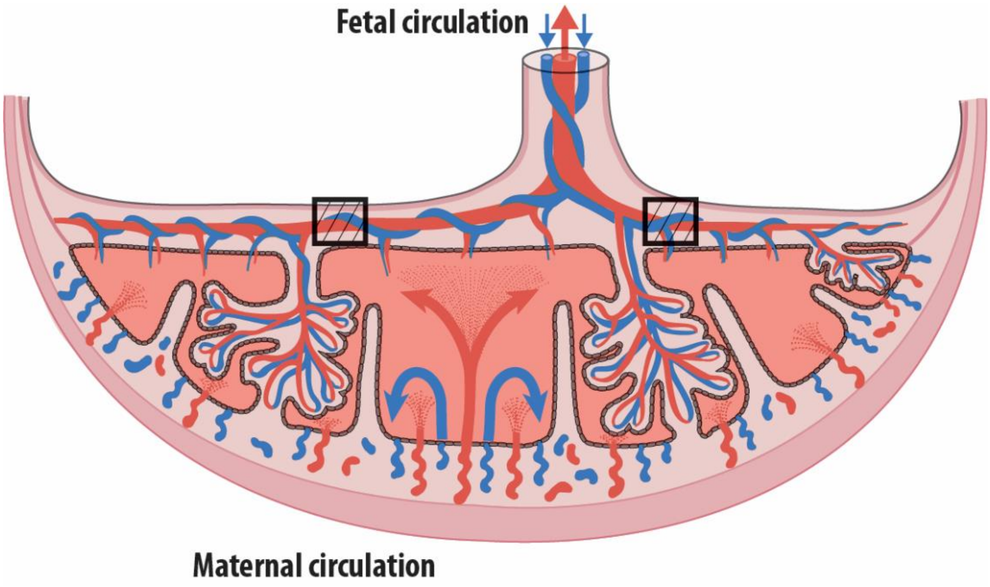

Supplementary table S1. Mean values of alpha diversity for all sample groups are divided by sample types. Metrics used: Shannon index and Observed OTUs. H: Kruskal-Wallis H test, p: p-value.

Sample group	Sample type	Shannon	H	p	OTUs	H	p
Placenta, water, unprocessed	Placenta	1.513	2.162	0.141	7	4.005	0.045
	Water	2.493			16		
Placenta, water, PMA	Placenta	1.691	1.207	0.272	11	2.335	0.127
	Water	2.426			14		
Placenta, water, dsDNase	Placenta	2.099	1.103	0.294	15	0.136	0.712
	Water	1.729			9		
Amniotic fluid, water, unprocessed	Amniotic fluid	2.324	0	1	18	0.027	0.869
	Water	2.445			14		
Amniotic fluid, water, PMA	Amniotic fluid	3.905	8.265	0.004	37	8.656	0.003
	Water	2.424			14		
Amniotic fluid, water, dsDNase	Amniotic fluid	2.180	0.095	0.758	20	0.182	0.670
	Water	1.597			8		
VD meconium, water, unprocessed	Meconium	3.139	2.175	0.140	47	12.15	0.001
	Water	2.395			13		
VD meconium, water, PMA	Meconium	2.598	0.210	0.646	56	10.82	0.001
	Water	2.138			12		
VD meconium, water, dsDNase	Meconium	2.422	1.357	0.244	48	12.24	0.001
	Water	1.673			7		
CS meconium, water, unprocessed	Meconium	2.737	0.892	0.345	22	1.238	0.266
	Water	2.395			13		
CS meconium, water, PMA	Meconium	2.983	2.232	0.135	33	2.672	0.102
	Water	2.138			12		
CS meconium, water, dsDNase	Meconium	2.806	6.531	0.011	18	4.237	0.040
	Water	1.673			7		

Supplementary table S2. Differentially abundant taxa of all placenta, amniotic fluid, and meconium samples against water, as well as meconium divided by the mode of delivery. The sample types are divided by laboratory treatment. Analyses used to determine the significance of differences in taxa abundances were ANCOM and the Mann-Whitney U test. P-value was used in the Mann-Whitney U test calculations to determine the significance with a threshold of $p < 0.05$.

	Species	ANCOM W	Mann-Whitney U, p-value
placenta vs water, unprocessed	Proteobacteria		0.010
placenta vs water, dsDNase	*Cutibacterium*	70	0.001
	Staphylococcus		0.004
amniotic fluid vs. water, PMA	*Micrococcus*	44	0.004
	Paracoccus		0.011
amniotic fluid vs. water, dsDNase	Actinobacteriota	11	0.003
	Cutibacterium	88	0.001
	Staphylococcus		0.048
meconium, delivery mode	Actinobacteriota		0.008
	Firmicutes	27	0.001
	Corynebacterium		0.044
	Lactobacillus	560	0.001
	Streptococcus		0.001
	Staphylococcus		0.008
	Escherichia-Shigella		0.001
Vaginal delivery meconium vs. water, unprocessed	Firmicutes		0.007
	Corynebacterium		0.019
	Vibrionimonas		0.002
	Lactobacillus		0.004
	Escherichia-Shigella		0.012
Vaginal delivery meconium vs. water, PMA	Bacteroidota		0.039
	Firmicutes		0.024
	Enterococcus		0.026
	Corynebacterium		0.015
	Lactobacillus		0.006
Vaginal delivery meconium vs. water, dsDNase	Firmicutes		0.010
	Corynebacterium		0.040
	Vibrionimonas		0.030
	Streptococcus		0.011
	Staphylococcus		0.001
	Clostridium		0.043
	Rhodobacter		0.043
	Escherichia-Shigella		0.001
C-section delivery meconium vs. water, unprocessed	Bacteroidota		0.036
	Vibrionimonas		0.010
	Ralstonia		0.032
C-section delivery meconium vs. water, dsDNase	Actinobacteriota		0.020
	Staphylococcus		0.019
